# Long-Term Anatomical and Hearing Outcomes of Canal Wall down Tympanoplasty for Tympano-Mastoid Cholesteatoma: A 20-Year Retrospective Study

**DOI:** 10.3390/life12111745

**Published:** 2022-10-31

**Authors:** Salvatore Ferlito, Ignazio La Mantia, Federico Merlino, Salvatore Cocuzza, Arianna Di Stadio, Giovanni Cammaroto, Ricardo Bartel, Gianluca Fadda, Giannicola Iannella, Quentin Mat, Stéphane Gargula, Justin Michel, Nicolas Fakhry, Antonino Maniaci

**Affiliations:** 1 Department of Medical and Surgical Sciences and Advanced Technologies “GF Ingrassia” ENT Section, University of Catania, 95123 Catania, Italy; 2 Department of Otolaryngology-Head and Neck Surgery, Morgagni Pierantoni Hospital, 47121 Forli, Italy; 3 Department of Otorhinolaryngology and Head and Neck Surgery, Hospital Universitario Mutua Terrasa, 08221 Barcelona, Spain; 4 Department of Otorhinolaryngology, San Luigi Gonzaga University Hospital, Università degli Studi di Torino, 10137 Turin, Italy; 5 Department of Sensory Organs, “Sapienza” University of Rome, 00100 Rome, Italy; 6 Department of Otorhinolaryngology, C.H.U. Charleroi, 6000 Charleroi, Belgium; 7 Faculty of Medicine and Pharmacy, University of Mons (UMons), 7022 Mons, Belgium; 8 Department of Otorhinolaryngology, Lariboisière Hospital, Assistance Publique–Hôpitaux de Paris, 75610 Paris, France; 9 Service D’otorhinolaryngologie et de Chirurgie Cervico-Faciale, Université Aix-Marseille, Hôpital de La Conception, 147, Boulevard Baille, 13005 Marseille, France

**Keywords:** tympanomastoid cholesteatoma, canal wall down mastoidectomy, residual disease, recurrence rate

## Abstract

Background: to evaluate the residual rate and the functional results after ten years from canal wall down tympanoplasty (CWD) for tympano-mastoid cholesteatoma. Methods: All the patients undergoing CWD for chronic otitis media with cholesteatoma at our ENT University Department between January 2002 and December 2022 were initially assessed. We performed clinical and diagnostic evaluation at baseline, 6 months, and then every year until an average follow-up of 10 years was obtained. Patients were consequently divided into two groups according to the presence (group A) or absence (group B) of the stapes superstructure. Results: After the selection process, 176 ears were included. The presence of the stapes superstructure was associated with better hearing outcomes (rate of patients < 30 dB) at 6 months (91.42% vs. 74.46%; *p* = 0.001) and 10 years (74.46% vs. 24.11%; *p* < 0.001). Residual cholesteatoma was reported in 10 ears, which included 2/35 ears in group A (5.71 %) and 8/141 in group B (5.67 %) (*p* = 0.993). The recurrent cholesteatoma rate was respectively 1/35 (2.85%) vs. 3/141 (2.18%) (*p* = 0.516). Conclusions: the CWD approach to cholesteatoma allows for effective long-term anatomical disease control and good hearing results when the stapes superstructure is preserved.

## 1. Introduction

The main goals of cholesteatoma surgery are eradication of the disease, prevention of recurrence, achievement of a dry, self-cleaning ear, and preservation of auditory function [1,2,3]. 

Various surgical techniques for the treatment of cholesteatoma have been described over the years with often conflicting anatomical and auditory results [3,4,5,6]. 

Although canal wall up tympanoplasty (CWU) is the preferred approach to preserve the anatomy of the tympanic cavity and maintain the functional ear, it is associated with a higher incidence of residual and recurrent cholesteatoma (up to 70%) [7,8].

Kerckhoffs et al., in a review yielding 2060 articles, reported six studies with greater disease recurrence after the CWU procedure [16.7–61.0%] compared to the CWD technique [0–13.2%] [8]. In particular, the authors demonstrated that CWU causes greater relapse of the disease compared to the CWD technique in adult patients with primary acquired cholesteatoma. However, many additional factors in patient care influence the best treatment decision, such as residual hearing and access to health care.

In contrast, tympanoplasty with canal wall lowering (CWD) has been shown to minimize residual and recurrent disease between 2% and 18%, but with lower levels of hearing [9,10,11,12,13,14]. 

However, hearing outcomes and rates of residual and recurrent disease in CWD surgery can be influenced by various factors, such as the degree of pathological involvement of middle ear structures, especially the presence or absence of the bracket superstructure [15,16,17,18,19,20,21,22,23,24]. 

Kim et al., in 2010 in a retrospective comparative study of 171 patients who underwent canal wall up or down mastoidectomy (CWUM and CWDM), did not report any significant difference (10.9 dB vs. 13.5 dB, respectively) (*p* = 0.21) in the postoperative ABG and patients’ rate with an ABG less than 20 dB (58.6% vs. 68.4%; *p* = 0.25). 

Britze et al., in a cohort of 147 cholesteatoma patients, observed significant improvements in all mean postoperative measures, with a mean one-year change in PTA of 5.47 dB (*p* = 0.003) and a mean change in intelligibility threshold of 5.94 dB (*p* = 0.002). In addition, the authors reported a mean air-bone gap (ABG) change of 4.05 dB (*p* = 0.26), with an increase in patients with ABG within 20 dB from 45% (*n* = 36) pre-operatively to 66% (*n* = 52) one year post-operatively.

Alternatively, Pareschi et al., in 2019, performed an analysis of functional outcomes and long-term prognostic factors of open tympanoplasty for the treatment of tympanomastoid cholesteatoma, including 895 consecutive patients with a follow-up of more than 10 years [24]. The authors reported 36.4% had a pure tone average (aPTA) ≤ 30 dB, with better outcomes at six months than in the long term. Risk factors associated with hearing deterioration included pediatric age and absence of bracket superstructure.

An interesting systematic review, which took up nine highly relevant articles, demonstrated, based on the results of the studies, a significantly higher mean ABG in patients with malleus present than in those with bracket absent (*p* < 0.001) and malleus status as a predictor of postoperative auditory outcome [25]. In addition, possible risk factors responsible for persistent conductive hearing loss after CWD include middle ear fibrosis, negative new cavity pressure, or displacement of ossicular grafts, which are associated with worse outcomes.

In this study, we performed a long-term retrospective analysis of patients who did not undergo CWD tympanoplasty to assess long-term rates of residual and recurrent disease and compare auditory outcomes.

## 2. Materials and Methods

### 2.1. Study Design and Patients

The “Strengthening the Reporting of Observational Studies in Epidemiology (STROBE)” guidelines were followed [26]. All the patients treated with CWD tympanoplasty for tympanomastoid cholesteatoma at our three ENT departments between January 2002 and December 2022 were initially recruited. Consequently, we selected patients reporting a minimum follow-up of 10 years for the analysis.

### 2.2. Inclusion and Exclusion Criteria

In order to evaluate the long-term outcomes of the CWD approach, the inclusion criteria were the following:

(1) Preoperative assessment including otomicrotoscopy, pure tone audiometry, middle ear CT scan
(2) Single-stage CWD mastoidectomy
(3) No previous otological surgery
(4) Age > 15 years old
(5) Minimum follow-up of at least 10 years

All the patients lost at follow-up or with incomplete data were excluded. Moreover, pediatric patients were not included due to their higher rate of recurrence of disease than adults and their poorer hearing outcomes as reported in the literature [22,27,28].

Patients with confirmed labyrinthine fistula at the time of surgery were not included because of possible sensorineural hearing loss before surgery and poor outcome.

### 2.3. Surgical Techniques

Surgical procedures were performed under general anesthesia, making a retro-auricular incision with a tympanomeatal flap. The CWD mastoidectomy was performed with the tympanomastoid structure well exteriorized obtaining a round-shaped cavity. The volume of the cavity was reduced with a tympanomeatal skin flap; thus, a meatoplasty was performed. The reconstruction of the tympanic membrane was performed using a temporal fascia graft with underlay technique. Alternatively, the reconstruction of the ossicular chain was performed with the remodeled autologous incus. 

### 2.4. Follow-Up Protocol

All patients underwent otoscopy and audiometric examination in the 6th month and every year for a minimum follow-up of 10 years. We have assessed the postoperative audiological outcomes according to the guidelines of the “Committee on Hearing and Balance” of the American Academy of Otolaryngology Head and Neck Surgery [29]. Pure-tone average air conduction thresholds (aPTA) at 500, 1000, 2000, and 4000 Hz were performed to assess postoperative audiological outcomes. The air-bone gap (ABG) was calculated for each patient.

Residual cholesteatoma was defined as persistent cholesteatoma in the middle ear or mastoid cavity following incomplete removal, while recurrent cholesteatoma was defined as cholesteatoma developing after complete removal [8]. Hearing and recurrence rates were also compared between subgroups according to the presence (group A) or absence (group B) of the stapes superstructure.

### 2.5. Statistical Analysis

Data analysis was performed using IBM SPSS Statistics for Windows, IBM Corp., released 2017, Version 25.0. IBM Corp. (Armonk, NY, USA)

We reported descriptive statistics on mean ± standard deviation or proportion. We assessed data normality using the Kolmogorov–Smirnov test of normality. The *t*-test for paired samples was used to determine the difference between observations. The Mann–Whitney U test was performed for comparisons. Multivariate analysis of variance (MANOVA) was performed to analyze the correlation of several dependent variables with long-term outcomes. The tests were two-tailed, and a *p*-value of < 0.05 was considered statistically significant.

## 3. Results

Amongst 396 ears treated for cholesteatoma, 176 ears were included: 105 were male and 71 were female. The mean age at surgery was 46.41 ± 10.1 years. The median follow-up was 11.78 ± 1.39 years. We found no stapes involvement in 35/176 (19.88%) patients (group A), while stapes superstructure disease was found in 141/176 (80.11%) patients with consequent autologous incus reconstruction (group B).

Facial nerve paralysis, vestibular symptoms, and cerebrospinal fluid leak have not been found in the selected case.

A total of 155/176 patients (88.06%) showed stable outcomes within 1 year after surgery, while 21/176 (21.94%) patients presented delayed re-epithelialization of the cavity due to granulation tissue formation and secretions. Of these 21/176, 10 patients required surgical revision; specifically, 4 subjects required revision to repair the neotimpan perforation while 6 patients required revision to widen the concomeatoplasty. 

Residual cholesteatoma was reported in 10/176 (5.68%) patients, while recurrent cholesteatoma occurred in 4/176 (2.27%) subjects (Table 1). 

Residual cholesteatoma was reported in group A in 2/35 (5.71%) patients localized in the mesotimpanus, whereas in group B it was reported in 8/141 (5.67 %) (*p* = 0.993). Among group B, in 6 cases it was localized in the mesotimpanus while in 2 cases in the anterior attic. Recurrent cholesteatoma from a new retraction pocket was reported in 1/35 (2.85%) patients in group A, and 3/141 (2.18%) cases in group B (*p* = 0.704).

Functional outcomes were reported according to each surgical patient subgroup (Table 2). 

**Figure 1:**
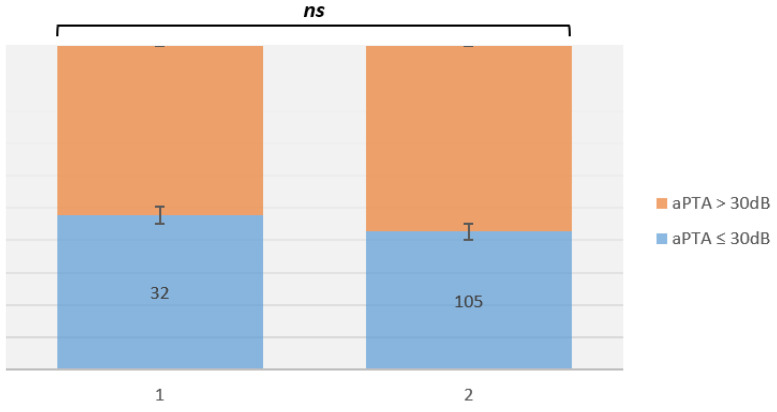
Abbreviations: ns, not significant. aPTA 6-month outcomes comparison after surgery among group A vs. group B. A significant difference was not found (*p* > 0.05).

At 6 months post-operative, when considering aPTA ≤ 30 dB, there were 137 (77.94%) patients: in group A there were 32/35 (91.42%), and in group B there were 105/141 (74.46%) (*p* = 0.457) (Figure 1). 

When considering aPTA > 30 dB, there were 39 (22.15%) patients: in group A there were 3/35 (8.57%), and in group B there were 36/141 (25.53%) (*p* = 0.070).

**Figure 2:**
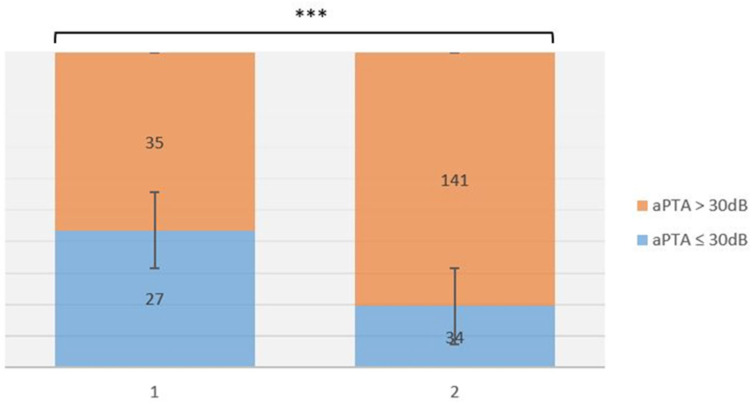
Abbreviations: ***, *p* < 0.001. aPTA 10-year outcomes comparison after surgery among group A vs. group B. A significant difference was found.

At 10 years after surgery, for patients with aPTA ≤ 30 dB, there were 61/176 (34.65%): in group A there were 27/35 (77.14%), and in group B there were 34/141 (24.11%). For patients with aPTA > 30 dB, there were 115/176 (65.34%): 8/35 (22.85%) in group A, while 107/141 (75.88%) patients were in group B (Figure 2).

The rate of patients with aPTA ≤ 30 dB HL at 10 years after surgery decreased significantly in both patient group A (91.42% vs. 77.14%; *p* = 0.001) and group B (74.46% vs. 24.11%; *p* < 0.001) (Table 2). 

Three patients (1.70%) underwent revision surgery for recurrent infection (late outcomes, >12 months). In addition, 4/176 (2.27%) patients presented with marked retraction (medialization) of the external pinna with obvious asymmetry when compared to the contralateral.

A chocolate cyst also called mucosa cyst retention occurred in 2/176 (1.13%) mastoid cavities that healed as a result of a collection of serum inside a mucosa pocket. Simple aspiration of the mucosa of the brownish serum reduced the size of the cyst, but its recurrence required the exposure of the cyst and the complete removal of the mucoperiosteal pocket.

In a multivariate analysis of long-term predictive factors for auditory outcomes, we found a significant correlation with stapes involvement (F = 38.958; *p* < 0.001) (Table 3).

In contrast, for both long-term residual outcomes and recurrence, otorrhea and perforation showed a significant correlation (*p* < 0.001 for all).

## 4. Discussion

Chronic cholesteatomatous otitis represents a particularly aggressive disease due to its different clinical course than chronic noncholesteatomatous otitis. The evolving character of cholesteatoma and osteolytic properties lead to relevant severe hearing complications [1,2,3]. Chronic cholesteatomatous otitis media presents with the symptoms of recurrent chronic otitis media, namely hearing loss, otorrhea, otalgia, and dizziness. The presence of cholesteatoma in the absence of chronic otitis media symptoms may also be asymptomatic. There are two types of cholesteatomas: congenital and acquired [6,7,8]. 

Congenital cholesteatoma is typical of pediatric age, results from the entrapment of embryonic epithelial remnants in the tympanic case, and manifests as a white retrotympanic mass (i.e., behind the tympanic membrane) found occasionally or diagnosed following hearing loss, in the absence of symptoms such as otorrhea or recurrent infections. Acquired cholesteatoma may result from chronic perforated otitis with invagination of the skin of the external ear canal within the tympanic case or from retraction of the tympanic membrane that becomes increasingly deep and in which keratin and epidermal scales accumulate.

In all cases, cholesteatoma could result in remodeling of the ossicular chain causing deafness or erosion of the bony structures that protect the facial nerve, meninges, and brain, predisposing to facial paralysis and meningo–brain infections [10,11,12,13,14].

Diagnosis requires a specialized ENT examination, including a careful history aimed at investigating the occurrence of recurrent episodes of otitis media. The objective examination consists of an otoscopy, performed by microscope or endoscope, through which it is possible to identify the cholesteatoma as a whitish mass behind the tympanic membrane, accompanied or not by a perforation in the membrane.

The main goal is to remove the cholesteatoma completely, ensuring ear reconstruction and adequate auditory outcomes. There are two types of surgery depending on whether or not the bone duct is preserved or reconstructed: bone duct conservative techniques involve a mastoidectomy or masto-antro-atticotomy with, usually, a posterior tympanotomy, referred to as closed techniques (CWUM). In contrast, techniques with bone wall sacrifice involving the emptying of cavities are called, again, "open technique tympanoplasties" or canal wall down procedures. 

The long-term outcome of the canal wall up (CWU) approach is nowadays considerably debated in the literature. Several factors influence the long-term outcomes of the CWD approach for tympano-mastoid cholesteatoma, both in regard to the effective eradication of the disease and restoring a satisfactory hearing function. 

Conflicting reports and opinions of surgeons have contributed to a long-standing debate on the merits of approaching cholesteatoma with an intact or a reduced canal wall. 

The CWD approach has been shown to minimize the rates of both residual and recurrent disease [23,24]. 

As early as 2013, Tomlin et al. identified how rates of recurrent and residual disease increased significantly when using a CWU approach rather than a CWD approach [8]. 

In addition, rates of recurrence following the typical two-stage intact canal wall operation were found to be comparable to a single-stage canal wall down operation.

Absolute indications for the CWD approach include unresectable disease, an unreconstructable posterior canal wall, failure of a first-stage canal wall up approach because of poor eustachian tube function, and inadequate patient follow-up [25,30]. 

The two different mechanisms of recidivism should be distinguished. Indeed, a residual cholesteatoma could occur due to the non-radically removed epidermoid cells as opposed to recurrence generated by a new retraction pocket containing keratin which subsequently develops cholesteatoma [8,31,32]. 

Kim et al. in their study in 2010 identified as prognostic factors for recurrence: preoperative middle ear granulation, atelectasis, or perforated pars tensa [2].

Further variables playing a role in the recurrence rate after the CWD approach are the creation of retraction pockets for insufficient lowering of the facial ridge and inadequate meatoplasty [30,31,32,33].

Vartiainen et al. reported how the same long-term results of surgical treatment varied according to the different cholesteatoma types detected, showing that hearing outcomes in attic and sinus cholesteatoma were significantly better than in pars tensa retraction cholesteatoma. The authors reported improved hearing in 36% of treated patients, concluding that surgery should be individualized according to the site and size of the cholesteatoma, but all large cholesteatomas would require a CWD procedure to avoid recurrence [28].

In our cases, the main cause of the recurrent cholesteatoma was the formation of retraction pockets for tubal dysfunction. Moreover, the posterior mesotympanum was the associated site of recurrent cholesteatoma found in our patients. 

The length of follow-up is also a recognized prognostic factor for functional stability outcomes [22]. 

Long-term follow-up of cholesteatoma cases is imperative for the detection of recurrent and residual disease, considering the average recurrence rate of CWD ranges from 2 to 18%, as reported in the literature [8,12,13,14].

In this regard, our study demonstrated the efficacy of the CWD approach both in the residual cholesteatoma rate of 10/176 (5.68%) and the recurrence rate of 4/176 (2.27%). According to the stapes superstructure involvement comparison, the residual rate was 2/35 cases (5.71%) in group A while there were 8/141 cases (5.67%) in group B, with no significant difference (*p* = 0.993). 

Furthermore, the long-term recurrent rate was found to be 4/176 cases (2.27%), of which, at subgroup analysis, 1/35 (2.85%) was from group A and 3/141 (2.18%) were from group B.

In multivariate analysis for long-term preoperative predictive factors influencing residual and recurrence rates, we found a significant correlation between preoperative otorrhea (F = 124.08, *p* < 0.001; F = 103.296, *p* < 0.001) and perforation (F = 92.643, *p* < 0.001; F = 52.848, *p* < 0.001) respectively. 

In the literature, the comparison between CWU and CWD approaches is still debated, especially when the ossicular chain is involved [10,14,27,28,31,32]. 

Aslan Felek et al. in a retrospective analysis of 134 adults with extensive acquired cholesteatoma treated with CWD surgery and ossicular chain reconstruction identified malleus handle and mucosal factors as important prognostic factors for hearing [12]. Indeed, the authors reported a postoperative ABG within 20 dB in 44% (*n* = 31) of patients with intact stapes superstructure versus 54% (*n* = 35) of patients absent stapes superstructure.

Kim et al. reported in 2010 no significant difference in the ABG ≤ 20 dB outcomes after type II ossiculoplasty between the CWU and CWD groups (58.6% vs. 68.4% respectively; *p* = 0.25) [2].

Okada et al. in 2013, in a series of cases, found the mean ABG was significantly smaller after type III tympanoplasty compared to type IV tympanoplasty, likely due to the increased severity of middle ear disease in patients undergoing type IV tympanoplasty with a missing stapes superstructure [33].

The need to reconstruct the ossicular chain in canal wall down tympanoplasty is frequently supported in the literature. Artuso et al. in 2004 evaluated functional outcomes at 2 years in a total of 60 patients undergoing canal wall down tympanoplasty (TPL CWD) and divided them into two homogeneous groups according to the ossiculoplasty performed (31 OCR vs. 29 non-OCR). The authors, at audiometry two years after surgery, revealed an aPTA of 42.98 dB in the OCR group and 58.65 dB in the non-OCR (*p* < 0.001), with a mean ABG of 24.06 and 35.54 dB respectively (*p* = 0.03) [17].

Alternatively, Gu et al. retrospectively evaluated the surgical outcomes of the CWD approach in 47 ears with chronic otitis media that had limited attic lesions with small, sclerotic, hypocellular mastoids [19].

The authors reported significant results for both the mean air conduction threshold, lowered from 37.2 ± 1.0 dB preoperatively to 32.8 ± 0.9 dB postoperatively (*p* < 0.01), and the mean ABG, reduced by 4.4 ± 0.4 dB (*p* < 0.01).

De Zinis et al. sought to identify factors associated with anatomical and functional outcomes of the CWD wall in 189 primary or recurrent cholesteatomas with a mean follow-up of 8 years, estimating predictive values of patient, disease, and surgical characteristics on cholesteatoma recurrence [30]. An overall postoperative ABG detected <20 dB was found in 30.7%, in which there were significant differences between intact or reconstructed vs. unreconstructed and eroded ossicular chains (43.9% vs. 13.4% respectively; *p* = 0.0001). Furthermore, the ABG was within 20 dB in 42.6% (46/108) when the mucosa of the tympanic cavity was normal and in 14.8% (12/81) when granulation tissue was present within the tympanic cavity (*p* = 0.0001). Long-term CWD mastoidectomy has also demonstrated an acceptably low complication rate in the literature. Kos et al. in a 7-year retrospective study of 259 cases reported an improved or at least unchanged overall hearing threshold in 72.0% of cases, while hearing loss occurred in 28% of cases, of which >29 dB occurred in 9.2% of cases [27]. Instead, a hearing loss >60 dB occurred in only 2 cases (0.7%) arising immediately after surgery. According to our data, the healing process leads to progressive deterioration of hearing. Indeed, the functional outcomes (aPTA < 30 dB) were significantly better at 6-month follow-up than at 10-year in both group A (91.42% vs. 74.46%) and B (77.14% vs. 24.11%) (*p* < 0.001). In multivariate analysis for long-term preoperative predictive factors influencing hearing outcomes, we found only stapes superstructure involvement as significantly correlated (F = 38.958, *p* < 0.001).

## 5. Conclusions

This retrospective multicenter study demonstrated that CWD tympanoplasty for tympanomastoid cholesteatoma could be an effective approach to achieve eradication of the disease at long-term follow-up, with a minimal residual rate. In patients with mesotimpanic or posterior wall cholesteatoma, tympanic membranes may be atelectatic due to negative intratympanic pressure and lead to disease recurrence. However, long-term auditory outcomes have not shown great stability at control aPTA, especially in patients without stapes superstructure compared with intact cases; instead, independent variables such as otorrhea and perforation could influence prognosis. 

## Figures and Tables

**Table 1:** Long-term residual/recurrent cholesteatoma rates comparison according to stapes superstructure involvement.

Features	Total (*n* = 176)	Group A (*n* = 35)	Group B (*n* = 141)	*p*-Value
Residual Cholesteatoma	10/176 (5.68%)	2/35 (5.71%)	8/141 (5.67%)	0.993
Recurrent Cholesteatoma	4/176 (2.27%)	1/35 (2.85%)	3/141 (2.18%)	0.516

**Table 2:** Hearing outcomes differed between stapes structures involvement at early and long-term follow-up. Group A, presence of stapes superstructure; Group B, absence of stapes superstructure.

Total (*n* = 176)				Group A (*n* = 35)			Group B (*n* = 141)		
	6 Months	10 Years	*p*-Value	6 Months	10 Years	*p*-Value	6 Months	10 Years	*p*-Value
≤30 dB	137 (77.84%)	61 (34.65%)	*p <* 0.001	32 (91.42%)	27 (77.14%)	*p* = 0.001	105 (74.46%)	34 (24.11%)	*p <* 0.001
>30 dB	39 (22.15%)	115 (65.34%)		3 (8.57%)	8 (22.85%)		36 (25.53%)	107 (75.88%)	

**Table 3:** Multivariate analysis on long-term predictive factors for hearing outcomes, residual and recurrence rate.

	Hearing Outcomes			Residual Rate			Recurrence Rate		
	Mean Square	F	Sig.	Mean Square	F	Sig.	Mean Square	F	Sig.
Age	0.305	1.396	0.239	0.004	0.019	0.89	0.757	3.508	0.063
Gender	0.003	0.013	0.909	0.073	0.305	0.581	0.056	0.235	0.628
Stapes involvement	5.256	38.958	<0.001	0.0003	0.002	0.964	0.008	0.049	0.826
Otorrea	0.001	0.012	0.914	4.306	124.08	<0.001	3.595	92.643	<0.001
Perforation	0.0004	0.01	0.922	3.192	103.296	<0.001	1.998	52.848	<0.001

## Data Availability

Not applicable.
